# From modern-day parasitology to paleoparasitology: the elusive past record and evolution of *Cryptosporidium*

**DOI:** 10.3389/fmicb.2023.1249884

**Published:** 2023-10-19

**Authors:** Kévin Roche, Frédéric Dalle, Nicolas Capelli, Romain Borne, Isabelle Jouffroy-Bapicot, Benoit Valot, Frédéric Grenouillet, Matthieu Le Bailly

**Affiliations:** ^1^UMR CNRS-UFC 6249 Chrono-environnement, University of Franche-Comté, Besançon, France; ^2^CNR LE Cryptosporidiosis Collaborating Laboratory, Santé Publique France, Dijon, France; ^3^Department of Parasitology/Mycology, University Hospital of Dijon, Dijon, France

**Keywords:** paleoparasitology, *Cryptosporidium parvum*, *Cryptosporidium hominis*, ELISA, ANCIENT DNA, sediment sample, coprolite, paleomicrobiology

## Abstract

Recent efforts have been made to review the state of the art on a variety of questions and targets in paleoparasitology, including protozoan taxa. Meanwhile, these efforts seemed to let aside *Cryptosporidium*, and we then intended to review its paleoparasitological record to assess its past distribution and favored detection methods, and eventually highlight needed research trajectories. This review shows that contrary to other parasites, most of the positive results came from South-American sites and coprolites rather than sediment samples, highlighting the need to test this kind of material, notably in Europe where many negative results were reported in the published literature from sediment samples. Moreover, aDNA-based detections are nearly absent from the paleoparasitological record of this parasite, though punctually shown successful. With their potential to address the evolutionary history of *Cryptosporidium* species, notably through their 18S rRNA tree, aDNA-based approaches should be encouraged in the future. In sum, and though the limits of currently used methods and materials remain unclear, this review highlights the potential role of coprolites and aDNA for the study of *Cryptosporidium* species in the past and how this history shaped their current diversity and distribution, notably among human populations but also farm animals.

## Introduction

Paleoparasitology aims to study the remains of parasites extracted from a variety of samples in archeological, paleontological, environmental or medical contexts. At the crossroads between humanities and life-sciences, it can address questions related to the evolution and past ecology of these parasites, but also to past human communities in which these parasites were circulating (in terms of health, hygiene, food habits or waste management for example). Since its beginning, this discipline focused on helminths, and to a lesser extent, protozoans, including *Cryptosporidium* (Le Bailly et al., 2021).

Ernest Edward Tyzzer identified *Cryptosporidium muris* in 1907 in the gastric glands of laboratory mice, although the Linnaean name was only formally established in 1910 (Tyzzer, 1907, 1910). Based on many morphological and life cycle details, Tyzzer later identified *C. parvum* on the basis of its smaller oocyst size and different niche, among other things, that is to say, the small intestine rather than gastric glands when transmitted to laboratory mice (Tyzzer, 1912). However, half a century went by before veterinary and medical scientists and practitioners showed any interest in it. Indeed, *Cryptosporidium* was shown to be associated with bovine diarrhea in 1971 (leading to economic loss), and several human cases were reported in 1976 by independent working groups (White, 2010). In 1982, several cases of severe protracted diarrhea were reported in the US in men and associated with Acquired Immune Deficiency Syndrome. This definitively triggered the interest of the medical community in this parasite (Fayer et al., 1990).

*Cryptosporidium* belongs to the phylum *Apicomplexa* alongside *Plasmodium* or *Toxoplasma*, agents of malaria and toxoplasmosis, respectively (Votýpka et al., 2017). *Cryptosporidium* spp. were classified within the *Coccidia* class, but as a result of recent evidence, including their newly resolved phylogenetic relationship through their 18S rRNA tree, ultra-structural similarities between *C. parvum* and gregarines, or their ability to develop their life cycle outside a host cell, they have now been reclassified in the sub-class *Cryptogregaria*, class *Gregarinomorphea*, that is to say a sister group of gregarines, rather than among coccidian parasites (Morrison, 2009; Hijjawi et al., 2010; Cavalier-Smith, 2014; Koh et al., 2014; Clode et al., 2015; Aldeyarbi and Karanis, 2016; Thompson et al., 2016).

Up until recently, the field of paleoparasitology mainly dealt with helminths (Nematodes, Cestodes, Trematodes and Acantocephalans). This is easily explained by the possibility to detect their microscopic eggs in archeological, paleoenvironmental, museum or paleontological samples from oviparous species (Le Bailly et al., 2021). Indeed, these microscopic eggs are resistant enough to be preserved through the millennia whereas soft tissue bodies tend to disappear quickly under taphonomical constraints (although mummy remains may preserve such bodies in rare circumstances). Moreover, these eggs can be recognized to the genus level (rarely to the species level) depending on their size, shape, ornamentation, or biological origin. Researchers have thus been looking for fossil or subfossil eggs under light microscopy since the early twentieth century (Ruffer, 1910).

When it comes to protozoans though, this is not a straightforward procedure, as even when producing oocysts, these parasites generally appear to be fragile, small, and lack any specific morphology. This is well exemplified by *Cryptosporidium parvum* oocysts, ranging from 4 to 6 μm, making them difficult to observe among environmental or fecal residues, and barely distinguishable from other micro-remains, such as fungal spores. Moreover, sample preparation for a microscopic approach includes a micro-sieving step (for example in the Rehydration–Homogenization–Micro-sieving protocol known as RHM protocol) during which the smaller mesh measures 20–25 μm, which is incompatible with the recovery of *Cryptosporidium* oocysts if the final flow through remains unchecked (Dufour and Le Bailly, 2013).

This does not mean that the paleoparasitology of protozoans is non-existent, far from it. Ten years ago, Frías and colleagues published an impressive review of the published literature on protozoans in ancient samples (Frías et al., 2013). Among other things, they shed light on the paleontological record of protozoans preserved in amber pieces. This is exemplified by a trypanosomatid of the genus *Paleoleshmania* observed in a female sandfly trapped in Cretaceous Burmese amber and proving the existence of these vector-borne parasites and Early Cretaceous interactions (Poinar and Poinar, 2004). This also encompasses apicomplexans (phylum to which *Cryptosporidium* spp. belong), as *Plasmodium dominicana* was recorded in Tertiary Dominican Republic amber. The ancient Apicomplexa record also includes intestinal species such as *Eimeria lonatoi* oocysts observed in much more recent deer coprolites, dated to 9,000 years BP in Brazil (Ferreira et al., 1992; Poinar, 2005). This record shows that paleoparasitological observations are not necessarily incompatible with the passing millennia, even when it comes to light microscopy-based diagnosis and protozoans.

More recently, the growing stock of data enabled Le Bailly and colleagues to contribute to this research and to review archeological occurrences of the human amoeba *Entamoeba histolytica* over the past 6,000 years (Gonçalves et al., 2004; Le Bailly et al., 2016). The authors showed that this protozoan had been circulating in Western Europe since at least the Neolithic period (5,700 years BP) and could have originated in the Old World before spreading toward the pre-Columbian Americas around the twelfth century. Interestingly, this review shows the importance of Enzyme Immunoassays (EIA), as nearly all reviewed papers used Enzyme-Linked immunoSorbent Assay kits (ELISA) to detect *E. histolytica* in ancient samples. This illustrates how molecular techniques can bypass microscopy limitations and complete classical approaches.

In the last 20 years, the contribution of aDNA-based methods to the field of paleoparasitology has greatly increased, including for protozoans (Côté and Le Bailly, 2017; Wood, 2018). One of the first papers focused on *Trypanosoma cruzi* aDNA extraction from 2000-year-old Chilean mummies (Madden et al., 2001). Since then, cutting-edge techniques have been applied on several occasions, notably for the recovery and comprehensive evolutionary and ecological history of *Plasmodium* spp. in humans (e.g., Gelabert et al., 2016; Marciniak et al., 2016) and other infectious pathogens (Arriola et al., 2020).

A rapid overview of protozoans in the paleoparasitological literature suggests that *Cryptosporidium* lags behind other targets despite its current importance in medical and veterinary domains. Consequently, in this paper, we intend to comprehensively review the topic, highlighting current research based on modern *Cryptosporidium* strains.

## Life cycle and epidemiology

*Cryptosporidium* is a single-celled parasite with a monoxenous life cycle, meaning that its whole development takes place in a single host before being transmitted to a new one. After sexual and asexual development, thin-walled oocysts excyst inside their current host, leading to autoinfection, while thick-walled oocysts spread in the environment through defecation and are transmitted to a new host (Bouzid et al., 2013). Transmission may occur via the fecal-oral route through the consumption of contaminated water or food, by human-to-human contact (anthroponotic), or non-human-animal-to-human contact (zoonotic) (Xiao, 2010). *Cryptosporidium* infects the gastrointestinal tract of a wide diversity of animal hosts, including human beings, but also other mammals, birds, reptiles and fish (Fayer, 2010; Zahedi and Ryan, 2020). The severity and form of symptoms can vary widely according to several factors, including host species, age, immunity, nutritional state, genetic background, infection site, or *Cryptosporidium* species and subtype (Flores and Okhuysen, 2009; Borad and Ward, 2010; Chalmers and Davies, 2010; Sponseller et al., 2014; Checkley et al., 2015). It is one of the main waterborne parasites across the world infecting humans due to its well-known resistance to basic water plant disinfectants, such as chlorination (Fayer et al., 1990; Baldursson and Karanis, 2011), but also as a result of the absence of efficient preventive or curative drugs (Dhal et al., 2022; Helmy and Hafez, 2022; Khan and Witola, 2023). *C. hominis* and *C. parvum* are the main strains currently known to infect human hosts. The former is more commonly found in developing countries than the latter although several other strains are known to cause zoonotic transmissions, such as *C. cuniculus* (Feng et al., 2018; Gilchrist et al., 2018; Zahedi and Ryan, 2020; Tichkule et al., 2021). With a global prevalence of 7.6% between 1960 and 2018 (Dong et al., 2020), *Cryptosporidium* is an important agent of diarrhea in humans and non-human animals, with noticeable impacts in terms of morbidity and mortality, primarily among immunocompromised people and children in low-income countries (Kotloff et al., 2013; GBD 2016 Diarrhoeal Disease Collaborators, 2018) but also in more developed regions (Costa et al., 2020) where its specific burden may be underestimated (McDonald et al., 2001; White, 2010) as well as its specific etiological agents (Robinson et al., 2008; Chalmers et al., 2009; Morris et al., 2019). Despite varying national surveillance strategies, notably in terms of genotyping efforts, *C. parvum* and *C. hominis* are shown to dominate among humans, with marked seasonality peaks of infection in late spring, late summer/early autumn in Europe (McLauchlin et al., 2000; Xiao, 2010; Cacciò and Chalmers, 2016).

## Paleoparasitological record of *Cryptosporidium* spp.

**Figure 1:**
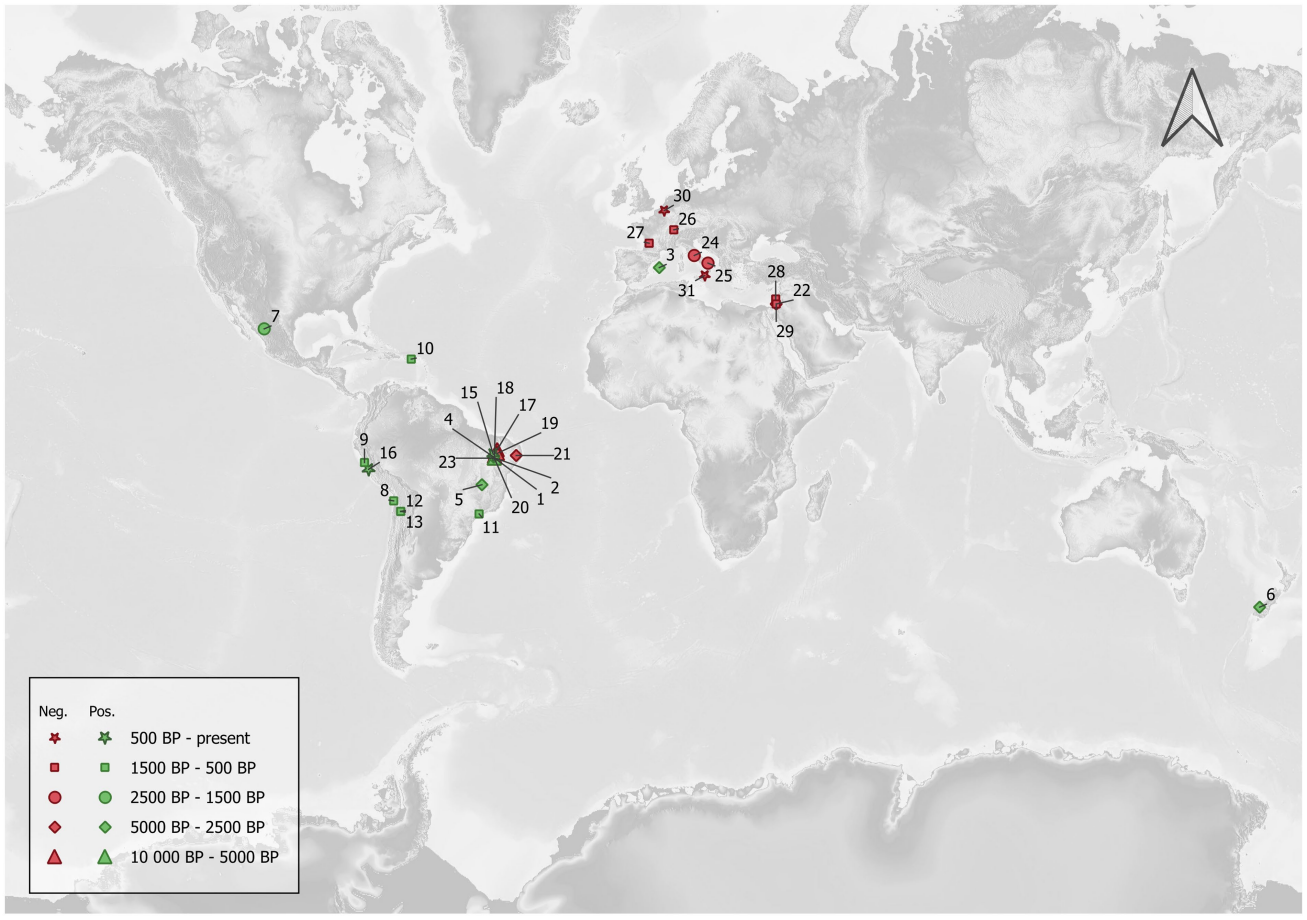
Archeological sites where *Cryptosporidium* detection attempts have been published. Nearly all positive sites are located in the New World, while negative ones are mostly located in the Old World. The correlation coefficient between the approximated central date in years BP and the ratio of positive samples for each site was negatively weak, suggesting a slightly decreasing ratio of positive samples with greater age, but this was not statistically significant (Kendall’s *τ* = −0.193; *p* = 0.149).

The map illustrated in Figure 1 documents detections of the past record of *Cryptosporidium* and reported negative results from the published literature. The mapping IDs of the archeological sites are reported in Tables 1, 2 with the corresponding site names, dating, sample types and the methods used for *Cryptosporidium* detection.

**Table 1:** Positive sites for *Cryptosporidium* detection.

Site and/or Region	Country	Period	Sample	Methods	Pos./Tot. samples	References	Map ID
Boqueirão da Pedra Furada, Piauí	Brazil	8,170 ± 80 BP	Rodent coprolite	ELISA	2/4	Leles et al. (2019)	1
Boqueirão da Pedra Furada, Piauí	Brazil	7,230 ± 80 BP	Human coprolite	ELISA	1/7	Leles et al. (2019)	2
Cova Estreta, Pollença, Serra da Tramuntana, Mallorca	Spain	4,950 ± 38 BP	*Myotragus balearicus* coprolite	ELISA	9/25	Borba Nunes et al. (2017)	3
Toca do Extrema II, Piauí	Brazil	4,730 ± 110 BP	Rodent coprolite	ELISA	1/1	Leles et al. (2019)	4
Gruta do Gentio II, Minas Gerais	Brazil	3,490 ± 120–430 ± 70 BP	Human coprolite	ELISA	1/10	Leles et al. (2019)	5
Andean region	-	500–3,000 BP	Human Mummified Feces	FIA / ELISA	15/39 and 8/15	Allison et al. (1999)	-
Dart River Valley, South Island	New Zealand	Late Holocene (<3,000 BP)	*Megalopteryx didinus* coprolite (upland moa)	PCR / Sanger	1/16	Wood et al. (2013)	6
La Cueva de los Muertos Chiquitos	Mexico	1,200–1,400 BP	Unidentified coprolites	ELISA	66/90	Morrow and Reinhard (2016)	7
Caserones Taparaca 40, Iquique	Chile	900 AC-800 CE	Human coprolite	ELISA	1/4	Leles et al. (2019)	8
PV35-4, North-central coast	Peru	770–830 CE	Human coprolite	FIA	1/22	Ortega and Bonavia (2003)	9
Sorcé, Vieques	Puerto Rico	1,400–540 BP	Human coprolite	WGA / Shotgun	1/9	Wiscovitch-Russo et al. (2020)	10
Sítio Fonseca, São Paulo	Brazil	1,010 ± 100 BP	Human sediment	ELISA	4/4	Leles et al. (2019)	11
San Pedro de Atacama, Antofagasta	Chile	940–1,240 CE	Human coprolite	ELISA	1/3	Leles et al. (2019)	12
San Pedro de Atacama, Antofagasta	Chile	900–1,450 CE	Human coprolite	ELISA	1/1	Leles et al. (2019)	13
Huayuri, Huánuco	Peru	1,200–1,400 CE	Human coprolite	ELISA	2/3	Leles et al. (2019)	14
Toca da Baixa dos Caboclos, Piauí	Brazil	400 ± 50 BP	Human coprolite	ELISA	1/1	Leles et al. (2019)	15
Vale do Rio Chillon, Lima	Peru	Late Inca Period	Human sediment	ELISA	1/2	Leles et al. (2019)	16

The mummies from “Andean region” (Allison et al., 1999) are not shown on Figure 1, thence do not have any Map ID number. Periods are indicated in years Before Present (BP) or Common Era (CE) according to the original publication.

**Table 2:** Negative sites for *Cryptosporidium* detection.

Site and/or region	Country	Period	Sample	Methods	Pos. samples/Neg. samples	References	Map ID
Sitio Antoniao, Piaui	Brazil	9,670 ± 140 BP	Rodent coprolite	ELISA	0/1	Leles et al. (2019)	17
Toca do Morcego, Bahia	Brazil	9,200 BP	Feline coprolite	ELISA	0/1	Leles et al. (2019)	18
Sitio do Meio, Piaui	Brazil	9,150 ± 60 BP	Feline coprolite	ELISA	0/1	Leles et al. (2019)	19
Toca dos Coqueiros, Piaui	Brazil	8,870 ± 60 BP	Canid, Feline and Human coprolites	ELISA	0/5	Leles et al. (2019)	20
Furna do Estrago, Pernambuco	Brazil	4,000–5,000 BP	Human coprolite	ELISA	0/2	Leles et al. (2019)	21
Jerusalem	Israel	2,700–2,500 BP	Latrine sediment	ELISA	0/4	Mitchell et al. (2023)	22
Toca de Cima dos Piloes, Piaui	Brazil	2,290 ± 60 BP	Rodent coprolite	ELISA	0/1	Leles et al. (2019)	23
Vacone	Italy	Roman Imperial-period	Drain fill sediment	ELISA	0/2	Ledger et al. (2021)	24
Vagnari	Italy	Roman Imperial-period	Drain fill sediment	ELISA	0/3	Ledger et al. (2021)	25
Chevenez	Switzerland	7th–9th c. CE	Human sediment	IFA/Immunochromatography	0/2	Le Bailly (2005)	26
Pineuilh	France	10th c. CE	Sediment	IFA/Immunochromatography	0/1	Le Bailly (2005)	27
City of Acre	Israel	12th–13th c. CE	Latrine sediment	ELISA	0/24	Mitchell et al. (2008)	28
Jerusalem	Israel	15th c. CE	Cesspool sediment and coprolites	ELISA	0/1 sediment & 0/8 coprolites	Yeh et al. (2015)	29
Brussels	Belgium	14th–17th c. CE	Latrine sediment	ELISA	0/7	Graff et al. (2020)	30
Sepulcher of the Priests of the Piraino Mother Church, Messina, Sicily	Italy	18th–19th c. CE	Intestinal content	ELISA / PCR-Sanger	0/1	Rollins et al. (2021)	31

As far as we know, the first mention of this protozoan appeared in the published literature in 1999. The authors looked for *Cryptosporidium* oocysts using Immuno Fluorescence Assay under the microscope (IFA), then Enzyme-Linked immunoSorbent Assay from 500 to 3000-year-old feces preserved in human mummies’ intestines from the Andean region in South America (Allison et al., 1999). Out of 39 individuals, 15 tested positive to *Cryptosporidium* oocysts under IFA and were subsequently tested with ELISA. Eight of these were finally confirmed. The archeological context is barely discussed in the paper, with references limited to the “Andean region” and very loose dating. Nevertheless, it did raise several important points: first, by showing that *Cryptosporidium* was not beyond the reach of paleoparasitology despite the fact that its oocysts are virtually undistinguishable in ancient samples under light microscopy alone; secondly, by showing that cryptosporidiosis must have plagued humans long before it appeared as a medical concern in the 1980s with massive and newly monitored epidemic events in the 1990s; and third, that *C. parvum* was certainly rife in pre-contact America, that is to say, before European colonization.

A few years later, a new paper reported *Cryptosporidium* detection in pre-Columbian Peru (Ortega and Bonavia, 2003). The authors screened 22 archeological feces (nearly always referred to as “coprolites” in archeological science literature) with IFA only. One of these feces collected on the P35-4 archeological site associated with the Peruvian Middle Horizon culture and dated to 770–830 CE was positive to *Cryptosporidium* sp. oocysts. This paper brought more evidence for pre-contact *Cryptosporidium* sp. in South America. Interestingly, the authors raised the question of surface antigen taphonomy and preservation over time and if any, their possible evolutionary change on a historical time scale. However, they did not have sufficient evidence to address the question.

**Figure 2:**
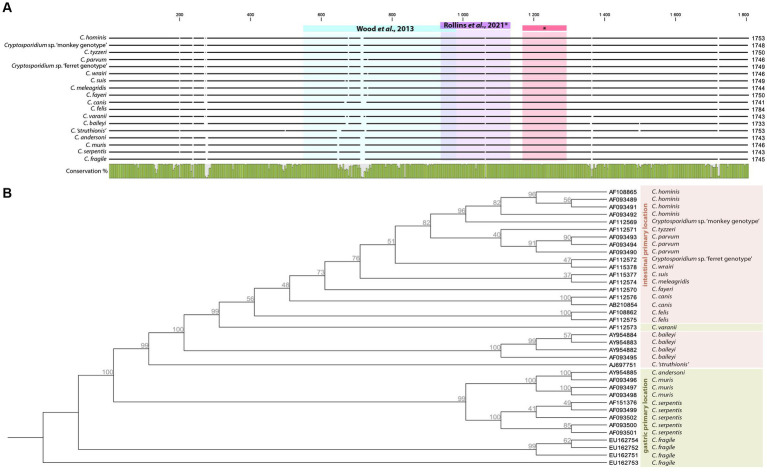
Alignment of selected complete 18S *Cryptosporidium* sp. sequences deposited on Genbank showing targeted regions in aDNA based papers **(A)**. Neighbor Joining tree with Jukes-Cantor measure in 1000 bootstrap replicates based on *Cryptosporidium* 18S complete sequences rooted on *C. fragile* (EU162753), primary infections sites are adapted from Šlapeta (2017) **(B)**.

Ten years later, *Cryptosporidium* was once again detected in ancient samples (Wood et al., 2013). The authors studied the parasitic diversity of several extinct moa in New Zealand through the light microscopy analysis of 84 well-preserved coprolites belonging to three distinct ratite bird species, and 16 were also analyzed with molecular analyses. The authors extracted the whole DNA from coprolite subsamples then amplified the obtained extracts using a previously designed primer set (Table 3) targeting a 350–400-bp-long fragment of the 18S gene, which is conserved in invertebrates (Figure 2A). The amplified extracts were subsequently cloned, and Sanger sequenced before being identified through BLAST alignments. One sequence successfully obtained from a single coprolite collected on the late Holocene Dart River Valley site, South Island, and associated with the extinct moa species *Megalopteryx didinus*, fell within the clade including the *Cryptosporidium* species, more specifically as a sister group to *C. ‘struthionis’*. This genotype is currently known to infect modern-day ostriches. The phylogenetic analysis later supported a ratite clade with the ostrich-infecting *Cryptosporidium* and basal to the two previously reported *Cryptosporidium* clades from other vertebrates. The authors suggested that it could hint toward the biogeography hypothesis of the Gondwanan Vicariance, not only documented through host divergence and isolation through time, but also through their parasites. The authors also raised the question of host–parasite coextinction phenomena, as *Cryptosporidium* was only identified from *Megalopteryx* in this study, but the scarcity of the sample set prevented further considerations (Wood et al., 2013). As subsequently noted, the long evolutionary *C. ‘struthionis’* branch would certainly be refined and split in light of novel, more geographically and temporally diverse isolates, including aDNA (Garcia-R and Hayman, 2016). This highlights the crucial role of aDNA and paleoparasitology in understanding the evolutionary history, current diversity and associations of hosts and parasites.

**Table 3:** Primer sets designed for the recovery of *Cryptosporidium* aDNA targeting different regions of its 18S gene.

Gene Target	Primer name	Primer seq.	Amplicon size (bp)	References
18S	Nem18SlongF Nem18SlongR	5′-CAGGGCAAGTCTGGTGCCAGCAGC-3′ 5′-GACTTTCGTTCTTGATTAATGAA-3′	350–400	Wood et al. (2013)
18S	–	5′-GCGAAACGATTTGCCAAGGA-3′ 5′-AGTTTCAGCCTTGCGACCAT-3′	195	Rollins et al. (2021)
18S	–	5′-GGAGCCTGCGGCTTAATTTG-3′ 5′-CCACCAACTAAGAACGGCCA-3′	127	Rollins et al. (2021)

In 2016, *Cryptosporidium* was again detected in human coprolites (Morrow and Reinhard, 2016). Ninety coprolites from the archeological site of La Cueva de los Muertos Chiquitos, in the Rio Zape Valley, Mexico, dated to 600–800 CE and associated with the Loma San Gabriel culture, were analyzed with ELISA to test for several protozoan parasites, including *C. parvum*. A total of 66 samples corresponding to a 73% detection frequency appeared as positive or likely to be positive to *C. parvum* in that study. It proved the presence of *C. parvum* in pre-contact Mesoamerica (previously shown in South America) and enabled the authors to discuss the pathoecology of *Cryptosporidium* among the ancient Loma San Gabriel people, in particular in terms of subsistence strategy and child mortality in light of previously obtained paleoparasitological and bioanthropological data from the same site. It also yielded the largest number of analyzed coprolites of human origin from a single archeological site to date. In this regard, the high detection frequency may raise some questions and legitimately triggered the authors’ interest (Morrow and Reinhard, 2016). Statistical analysis did not show any spatial association of positive samples with percolation and leaching phenomena in the different archeological squares and strata. Furthermore, the authors took care to cold conserve their samples to avoid fungal and bacterial growth that may lead to cross-reactions in coproantigen-based assays and ultimately to false positives (Morrow and Reinhard, 2016). These questions mirror those raised by Ortega and Bonavia (2003), not only regarding antigen stability, taphonomy and evolution through time leading to false negatives, but also their obliteration in cross-reaction phenomena, leading to false positives, and call for a closer monitoring of this matter in the future.

In 2017, 25 coprolites from *Myotragus balearicus* were screened for the presence of *C. parvum* using ELISA tests (Borba Nunes et al., 2017). *M. balearicus* is an extinct Caprinae endemic to the Eastern Balearic Islands. The source material was collected from Cova Estreta, Pollença, Serra a Tramuntana, Mallorca, and dated to 4,950 ± 38 BP. Among the sample set, nine samples tested positive for *C. parvum*. This paper brought up several important points. First of all, it was, and as far as we know, remains to date the only paleoparasitological detection of *Cryptosporidium* in Europe. Secondly, it echoes previously cited work testing for parasites of extinct moas in New Zealand (Wood et al., 2013), raising the same questions about coevolutionary and coextinction process between hosts and parasites during the Holocene. Finally, it also showed that ELISA tests were a functional tool in this matter too, as Wood and colleagues looked for aDNA alongside microscopy, but did not employ any EIA.

More recently, 60 samples consisting of anthropogenized sediment or preserved human feces were collected from 15 archeological sites in South America (Leles et al., 2019). The sites are located in Brazil, Chile and Peru, ranging from 9,800 ± 80 years BP to 400 ± 50 years BP, that is to say an extensive part of the Holocene encompassing pre-and post-contact chrono-cultural contexts. Most sites yielded human coprolites, but a couple also yielded human sediment, canid, feline, and rodent coprolites. ELISA tests were used to detect *Cryptosporidium* in the samples alongside other protozoans. Sixteen samples appeared positive to *Cryptosporidium* sp., corresponding to a 26.6% detection rate in the sample set. The oldest positive samples come from human and rodent coprolites from the same archeological site and were dated to 7,230 ± 80 BP. Interestingly, the authors critically tested the yields of immunoenzymatic techniques through the comparison of results obtained with microscopy, ELISA, IFA and PCR. This experimental approach was based on *Giardia duodenalis,* which was detected in 48 samples (46.6%). In spite of the high detection frequency among archeological samples using immunoenzymatic tests, the authors could not amplify any *G. duodenalis* sequences, even though specifically designed primer sets were previously shown to work on modern and experimentally taphonomized feces. According to the authors, this discrepancy raises the question of the specificity of immunoenzymatic tests for archeological materials and necessitates further studies (Leles et al., 2019). Their paper echoes previously raised questions and adds experimental data.

At last, 9 coprolites from Puerto Rican indigenous cultures in Vieques and dated to 540–1,400 years BP were analyzed through light microscopy and metagenomic shotgun sequencing (Wiscovitch-Russo et al., 2020). While microscopy allowed the recovery of several helminth taxa, non-targeted whole genome amplification (WGA) and sequencing allowed the recovery of one sequence latter associated to *Cryptosporidium* spp. HSP70 protein coding gene through BlastX and phylogenetic analysis. Interestingly this paper confirmed the need of integrative approach previously suggested by Côté et al. (2016) as aDNA and microscopy-based data did not overlap. The authors subsequently used network modeling to recreate parasite–host interactions in conjunction with zooarcheological data and highlighted the crucial role of rodents and canids in the transmission of zoonotic parasites among ancient Huecoid and Saladoid communities that co-existed at the Sorcé Site. Meanwhile, *Cryptosporidium* was not included in this analysis due to the lack of accuracy of the sequence. Though shotgun-based approaches are commonly used for the study of ancient intestinal microbiomes (e.g., Hagan et al., 2020; Jacobson et al., 2020), protozoa like *Cryptosporidium* may be flooded within the bacterial signal thence leading to false negative or unspecific results.

Several points emerge from these papers showing positive results for *Cryptosporidium* in the past. First, to our knowledge, only seven papers mention positive results for 15 archeological or paleontological sites (Table 1). Moreover, 13 sites are located in South or Meso-America, while the remaining ones (*N* = 2) are in Europe and Oceania, respectively (Figure 1). Coprolites were the main materials for the recovery of *Cryptosporidium* on these 15 archeological sites. South and Meso-American sites are mostly dated to the pre-contact period, ranging from 8,170 years BP to the late Inca period. European and Oceanian sites are dated to 4,950 years BP and the late Holocene (<3,000 BP) respectively. On the other hand, we found eight papers reporting negative results on 15 archeological sites, six of which are in Brazil (ranging from 9,670 to 2,290 years BP), while nine sites are in the Old World (Italy, Switzerland, Belgium, France and Israel, ranging from the Iron Age to the nineteenth century) (Figure 1). Among these negative reports, coprolites and sediment were indicated as the source material in eight and eight analyses, respectively, (one site used both). In both groups (positive and negative sites), ELISA was the main diagnostic tool, complemented with IFA and PCR/Sanger sequencing on rare occasions (Table 2).

This contrasts with the reviewed literature regarding *E. histolytica* (Le Bailly et al., 2016). Indeed, Le Bailly and colleagues identified 27 archeological sites, most of which (*N* = 19) were located in the Old World, more specifically in Europe (Switzerland, Greece, France, Belgium, Italy, Latvia and Israel), while a minority (*N* = 8) were located in the New World (USA, Guadeloupe, Chile, Peru, Argentina). We could suggest that light microscopy detection leads to more observations of *E. histolytica*, yet only two papers mentioned light microscopy while all the other diagnoses were based on ELISA tests. These results could also be imputed to the fact that paleoparasitologists’ interest in *E. histolytica* began earlier than their interest in *Cryptosporidium*. However, while the first paper regarding the latter was published in 1999, only two earlier papers focus on *E. histolytica* (Witenberg, 1961; Fouant et al., 1982; Allison et al., 1999). This situation may also imply that Old World researchers or those working with Old World materials, are not interested in *Cryptosporidium,* and that consequently this protozoan is under explored in the literature and virtually absent in the past record. But this is refuted by the published literature mentioning many failed attempts to detect *Cryptosporidium* in the Old World (Le Bailly, 2005; Mitchell et al., 2008; Yeh et al., 2015; Graff et al., 2020; Ledger et al., 2021; Rollins et al., 2021; Mitchell et al., 2023), to which we can add our own unpublished negative results. Overall, these attempts employ similar methods to those used for positive results, mainly ELISA tests, with a few exceptions. Moreover, other gastrointestinal parasites, including protozoans, are systematically detected with ELISA (e.g., Mitchell et al., 2023; Table 2). Therefore, we can suggest that a generally smaller number of tested samples in the Old World and/or taphonomical constraints explain this asymmetrical record.

**Figure 3:**
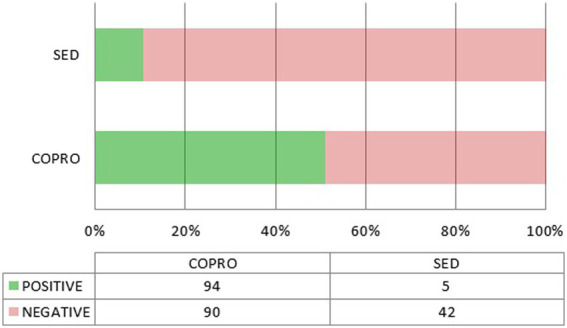
Positive and negative outcomes depending on the source material (coprolite or sediment). Only ELISA tests are included.

It is important to underline that nearly all the positive results for *Cryptosporidium* come from coprolites (Figure 3). Interestingly, this is also true for the only two positive results from outside the Americas, namely *M. balearicus* coprolites from the Balearic Islands in Spain (Western Mediterranean), and *M. didinus* coprolites from New Zealand (Wood et al., 2013; Borba Nunes et al., 2017), both from extinct species. In the former, *C. parvum* was identified through ELISA testing, and a fragment of its 18S gene was sequenced from the extracted aDNA in the latter. A chi-square test of independence was applied to the whole data set compiled for this review to examine the relationship between sample type (coprolite or sediment) and *Cryptosporidium* detection (positive or negative). Coprolites were significantly associated with a higher detection frequency, both when considering all performed tests (ELISA, IFA, and PCR), χ^2^(1, *N* = 305) = 17.2499, *p* < 0.01, and ELISA tests only, χ^2^(1, *N* = 231) = 25.0108, *p* < 0.01.

The association between sample type and detection outcome would appear to confirm that coprolites are indeed a better material for the recovery of *Cryptosporidium*. However, this is not so clearcut as most of the sediment samples were collected from skeletal pelvic areas or latrines/cesspools, and thus highly spoiled with fecal residues. But we must be extremely cautious as the sediment sample set is very small (*N* = 50; 47 when considering ELISA only) and sampling bias cannot be excluded.

Overall, immunodiagnostic-based papers report negative and positive controls following manufacturers’ standards, lending credibility to the positive and negative results presented in Tables 1, 2. Meanwhile, it cannot totally exclude taphonomic biases as discussed by Leles et al. (2019). On the other hand, aDNA-based approaches follow stringent protocols from the field of paleogenetics, including extraction blanks. When using newly designed primer sets for *Cryptosporidium* though, it is not always clear if they were tested and validated on fresh and/or experimentally taphonomized materials beforehand as described by Leles et al. (2019) regarding *G. intestinalis*. This can make negative results more difficult to interpret, as shown in the study by Rollins et al. (2021), but ELISA tests tended to confirm them.

The previously mentioned possibility of false positive results when using coproantigens from coprolites would not be an issue for aDNA-based results, as published by Wood et al. (2013). But aDNA-based tests only account for a tiny minority of the whole sample set. Moreover, on at least two occasions, sediment materials also yielded positive results in the Americas, namely from the Sítio Fonseca, São Paulo, Brazil, dated to 1,010 ± 100 BP, and from the Vale do Rio Chillon site, Lima, Peru, dated to the Late Inca period (Leles et al., 2019). Coprolites appear to be more prone to *Cryptosporidium* detection on the one hand, but on the other hand, the use of sediment material does not seem to preclude such detection. These results may be explained by several factors regarding taphonomic conditions in coprolites and sediment samples: (1) remains of parasites are much more diluted in sediment samples compared with coprolites consisting in discrete specimens of concentrated fecal material in stratigraphic units; (2) latrines and cesspools from which originate many negative sediment samples may be particularly subjected to continual degradation due to biological taphonomic agents like fungi, mites, and nematodes as suggested by Reinhard et al. (2019) compared with individual coprolites quickly buried and not triggering the same amount of biological activity. Furthermore, the detection of *Cryptosporidium* may be due to wider favorable conservation conditions at the local site, the same that actually favored the preservation of coprolites in the first place and explain the vast published literature on this topic from American sites (Shillito et al., 2020).

A few key points seem to emerge for future research. First of all, it is essential to build on previously asked questions about coproantigen sensitivity and specificity in archeological materials. Secondly, larger sample sets and coprolites from the Old World now need to be explored. But coprolites can be harder to find in Europe than in other regions of the world with varying conservation conditions, and could be replaced by human mummified intestinal tissues, known to occur in a wide variety of chrono-cultural contexts around the Mediterranean. Indeed, while we expect to find only thick-walled oocysts released in coprolites, intestinal tissues may still carry thin-walled oocysts, trophozoites and other stages of the previously mentioned asexual and sexual *Cryptosporidium* life cycle infecting numerous enterocytes. Not only can these stages be theoretically observed through SEM, but the abundance of genetic material may also facilitate aDNA detection. Thirdly, the use of molecular tools must be developed to complement immunoenzymatic tests. The latter are fast and easy to set up, but they are designed for fresh stool samples rather than archeological materials and restricted to a narrow species spectrum. On the other hand, aDNA-based methods have a long track record of archeological-like materials adaptation and allow catching a wide genetic diversity including unknown or extinct taxa, but necessitate deeper and time consuming designs from bioinformatics to wet lab experiments. Indeed, only two papers mentioned aDNA-based targeted attempts to detect *Cryptosporidium* from archeological or paleoenvironmental materials (Wood et al., 2013; Rollins et al., 2021). Both papers targeted the 18S gene thereby facilitating aDNA recovery. The 18S copy number (20 per oocyst) makes it an appropriate candidate for aDNA extraction considering that *Cryptosporidium* reductive evolution deprived it of high copy number mitochondrial DNA. Several primer sets were designed and tested with ancient samples (Table 3), both targeting hyper variable regions catching a wide variety of species, and highly conserved ones (Figure 2A). Moreover, a number of primer sets targeting the *Cryptosporidium* 18S gene were independently designed for the analysis of modern samples, some of which would certainly be suited to aDNA recovery based on their short PCR product (not reported in this paper). Last but not least, 18S allows evolutionary relationship inferences among *Cryptosporidium* species and genotypes, showing clustering according to primary infection location and host species (Figure 2B). It would be extremely interesting to include ancient *Cryptosporidium* 18S genes in this tree, be it from extinct or still extant strains.

**Figure 4:**
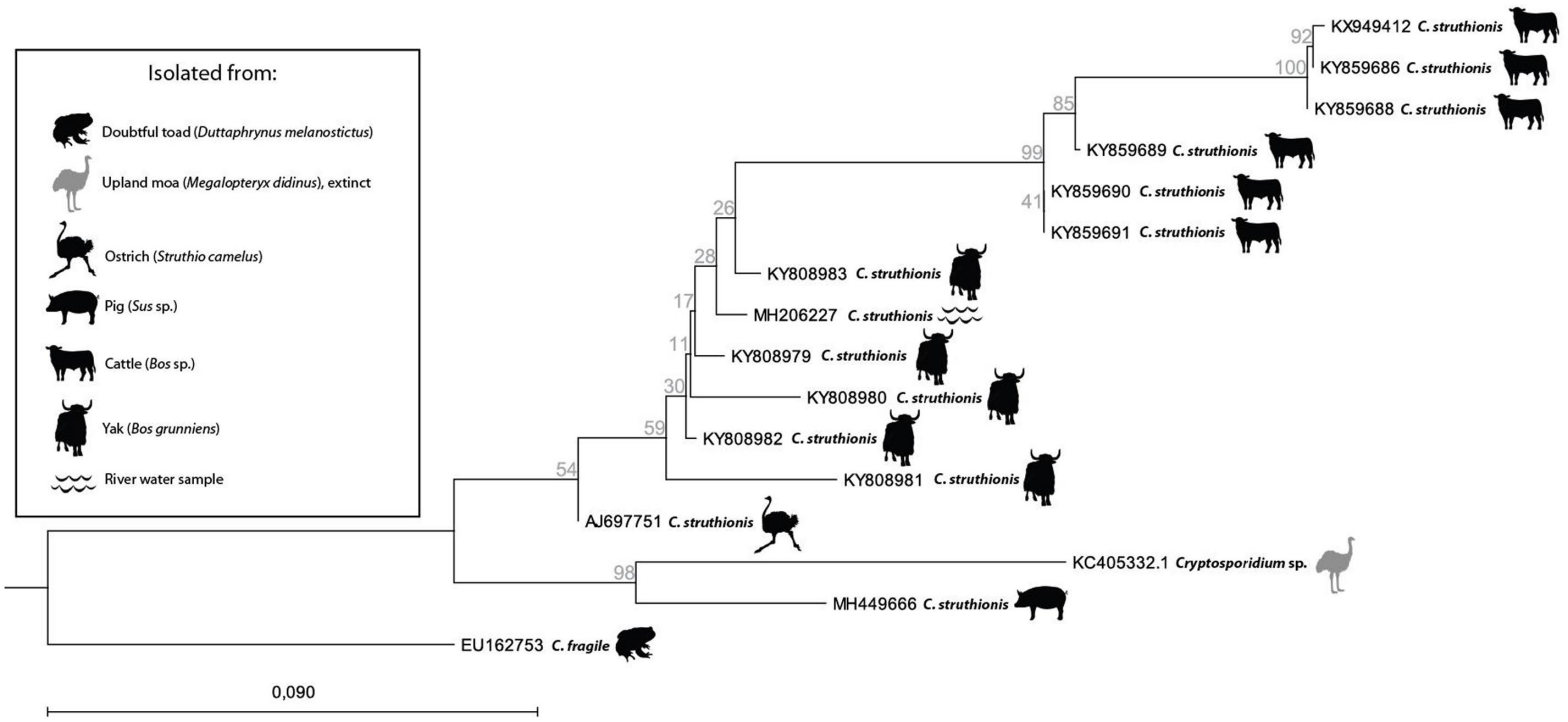
Neighbor Joining tree with Jukes-Cantor measure in 1000 bootstrap replicates based on *Cryptosporidium ‘struthionis’* 18S partial sequences deposited on Genbank and rooted on *C. fragile* (EU162753). Icons show animal hosts or environmental samples from which *Cryptosporidium* was isolated.

As mentioned above, aDNA fragments of *Cryptosporidium* were extracted from extinct flightless birds in New Zealand (Wood et al., 2013). Interestingly, modern *C. ‘struthionis’* partial sequences subsequently deposited on Genbank tend to be from farmed mammals (yak, cattle, and pig) in the Qinghai-Tibetan Plateau Area, China (Figure 4). While isolates tend to form distinct clades depending on their host (respectively yak or cattle), the ancient moa sequence from New Zealand clustered with a modern farmed pig isolate, but remained a sister group of the originally identified ostrich isolate (Figure 4). This should not come as a surprise as *Cryptosporidium* species and genotypes were reported in modern isolates from at least 150 mammal species and 30 bird species, with some very broad host spectrums (Fayer, 2010). Without any doubt, adding ancient sequences, both from farm animals recovered in archeological deposits, and from extinct wildlife, will benefit our understanding of the spread of parasites and host adaptation through time. The need of documenting ancient sequences of *Cryptosporidium* is also highlighted by recent works based on modern genomes. These works suggested that after initial diversification following the Cretaceous-Paleogene transition (K-Pg boundary), past human activities in historical times (as transatlantic migrations and sanitation development) may explain the current diversity and distribution of this parasite among humans (Garcia-R and Hayman, 2016; Nader et al., 2019; Cabarcas et al., 2021; Tichkule et al., 2022). These hypotheses may be tested through aDNA recovery as illustrated in the case of other infectious agents as *Mycobacterium tuberculosis* (Brynildsrud et al., 2018). Furthermore, other works including eukaryotic pathogens highlighted the interest to obtain ancient DNA to better understand not only the past distribution of currently neglected infectious agents, but also their molecular evolution including pathogenicity factors over time, or their population genomics up to the present days (Spyrou et al., 2018; Doyle et al., 2022).

## Conclusion

In this paper, we aimed to review the published literature regarding *Cryptosporidium* detection in the past. We showed that positive detections do exist but are rarer when it comes to *Cryptosporidium* compared to other parasites, including protozoans such as *E. histolytica*. Nonetheless, the past record of *Cryptosporidium* is well attested, mainly in the pre-contact Americas. Very few positive results have emerged from European and more recent archeological sites, but this may be due to small sample size and source material (sediment rather than coprolites), as coprolites appear to be a better source material for the recovery of *Cryptosporidium*. Nearly all reports, positive and negative, used ELISA tests. aDNA-based methods may certainly complement *Cryptosporidium* detection methods, expand the currently known past record, and participate in a wide array of questions, be it in terms of parasitology, evolutionary, or archeological sciences. Such methods are strategically located at the crossroads of those disciplines, and could potentially relate the emergence of pathogenic strains to past human activities, as previously suggested by modern-day genome studies, shed light on their development and evanescence, and enhance our understanding of their current distribution and diversity.

## Author contributions

KR and MLB conceived the manuscript. KR wrote the manuscript and conceived the figures. FD, NC, RB, IJ-B, BV, and FG critically reviewed and edited the final manuscript. All authors contributed to the article and approved the submitted version.
